# Carrier-Mediated and Energy-Dependent Uptake and Efflux of Deoxynivalenol in Mammalian Cells

**DOI:** 10.1038/s41598-017-06199-8

**Published:** 2017-07-19

**Authors:** Xiaoming Li, Peiqiang Mu, Jikai Wen, Yiqun Deng

**Affiliations:** 1 Guangdong Provincial Key Laboratory of Protein Function and Regulation in Agricultural Organisms, College of Life Sciences, South China Agricultural University, Guangzhou, Guangdong 510642 P.R. China; 2 Key Laboratory of Zoonosis of Ministry of Agriculture, South China Agricultural University, Guangzhou, Guangdong 510642 P.R. China

## Abstract

Deoxynivalenol (DON) is one of the most abundant mycotoxins and exerts many adverse effects on humans and animals. To date, the transporting mechanism of DON in mammalian cells remains unclear. In this study, the parallel artificial membrane permeability assay (PAMPA), Transwell models and metabolic inhibitors were used to determine the possible transporting mechanisms of DON in Caco-2, MDCK and HepG2 cells. PAMPA and Transwell models showed reduced passive transport and increased intestinal absorption, indicating a carrier-mediated transporting mechanism. Furthermore, higher unidirectional transport of DON was observed in the basolateral-to-apical direction than in the apical-to-basolateral direction, indicating the existence of efflux proteins. Interestingly, DON was accumulated in the nucleus, and no DON was detected in mitochondria, indicating that the nucleus may be the main target organelle of DON. Moreover, the use of various transporter inhibitors in different cells shows that organic anion transporters, organic cation transporters, and organic anion-transporting polypeptides participate in DON uptake, and P-glycoprotein is the major efflux protein. Importantly, DON uptake is strongly inhibited by metabolic inhibitors and is highly dependent on temperature. In summary, carrier-mediated and energy-dependent uptake and efflux mechanisms for DON in mammalian cells are reported, aiding in improving our understanding of its toxicological mechanisms.

## Introduction

Deoxynivalenol (DON) belongs to a group of type B trichothecenes that are mainly produced by *Fusarium graminearum* and *Fusarium culmorum*, which predominantly infect grains such as wheat, barley, oats, rye, and maize throughout the world^1–4^. DON is also called vomitoxin because it induces vomiting^5, 6^. DON exerts various toxic effects on humans and animals, such as growth inhibition, reduced nutrient absorption, the formation of intestinal lesions and immunosuppression^7, 8^. DON contamination is difficult to control and has become a substantial threat to animal production and food safety.

The widely accepted mechanism of DON toxicity is that it binds to ribosomes, thus affecting the activity of peptidyl-transferases to inhibit translation and causing serious toxic effects^9^. Moreover, the mitochondria and nucleus have also been shown to play important roles in DON toxicity^10^. DON must first enter cells to exert its toxic effects. Nevertheless, the relationship between DON toxicity and absorption is still unclear. DON was not able to enter cells in some studies^9^, but directly entered cells and was transported to the target organelles, such as ribosomes, the mitochondria and nucleus, in other studies^10, 11^. Therefore, we must clarify the mechanisms of DON transport to understand the mechanisms underlying DON toxicity.

The transport of DON was summarized previously and almost all possible mechanisms, including free diffusion, transmembrane transport and bulk-phase endocytosis/pinocytosis, were proposed, because evidence was lacking and inconsistent data were reported in different cases^9^. Several studies showed that DON can reduce the integrity of intestinal barriers and cross the barrier through paracellular transport or passive diffusion in human, chicken and porcine intestine cells^12–16^. However, how DON enters cells without disintegrating cell membranes and how DON diffuses across the cell membrane, overcoming its high polarity, remain unknown. Interestingly, one report showed that P-glycoprotein and MRP2 were involved in DON efflux in Caco-2, LLCPK1 and MDCK II cells^17^, and another report showed that T-2 toxin, which is a type A trichothecene, was uptaken by OATs and OCTs^18^. These studies suggest that there may be a transporter involved in DON uptake^14^. Thus, the transport of DON in mammalian cells remains unclear and need further investigation.

Transporters, including ATP-binding cassette (ABC) family transporters, organic anion transporters (OATs), organic cation transporters (OCTs) and organic anion-transporting peptides (OATPs), play important roles in the uptake and efflux of exogenous compounds and are distributed in different tissues in a subtype-selective manner^19^. ABCB1 and ABCC2 have been reported to be the major transporters involved in the efflux of DON and nivalenol in Caco-2 and MDCK II cells^17, 20^. However, DON transport was not affected by P-glycoprotein (PgP) or ABCC2 inhibitors in Caco-2 cells^16^. Therefore, further clarification of the transporters involved in DON uptake and efflux is required.

In this study, the widely used model cell lines in drug absorption research, Caco-2, HepG2 and MDCK^21, 22^, were chosen as the representative cells from the intestine, liver and kidney, respectively, and three major approaches, the parallel artificial membrane permeability assay (PAMPA), Transwell membrane models and transporter inhibitors, were used to intensively study DON uptake and efflux. This study clarified the mechanism of DON transport in mammalian cells and might be valuable for designing methods for reducing DON induced injuries to cells.

## Results

### Cytotoxicity of DON in Mammalian Cells

**Figure 1:**
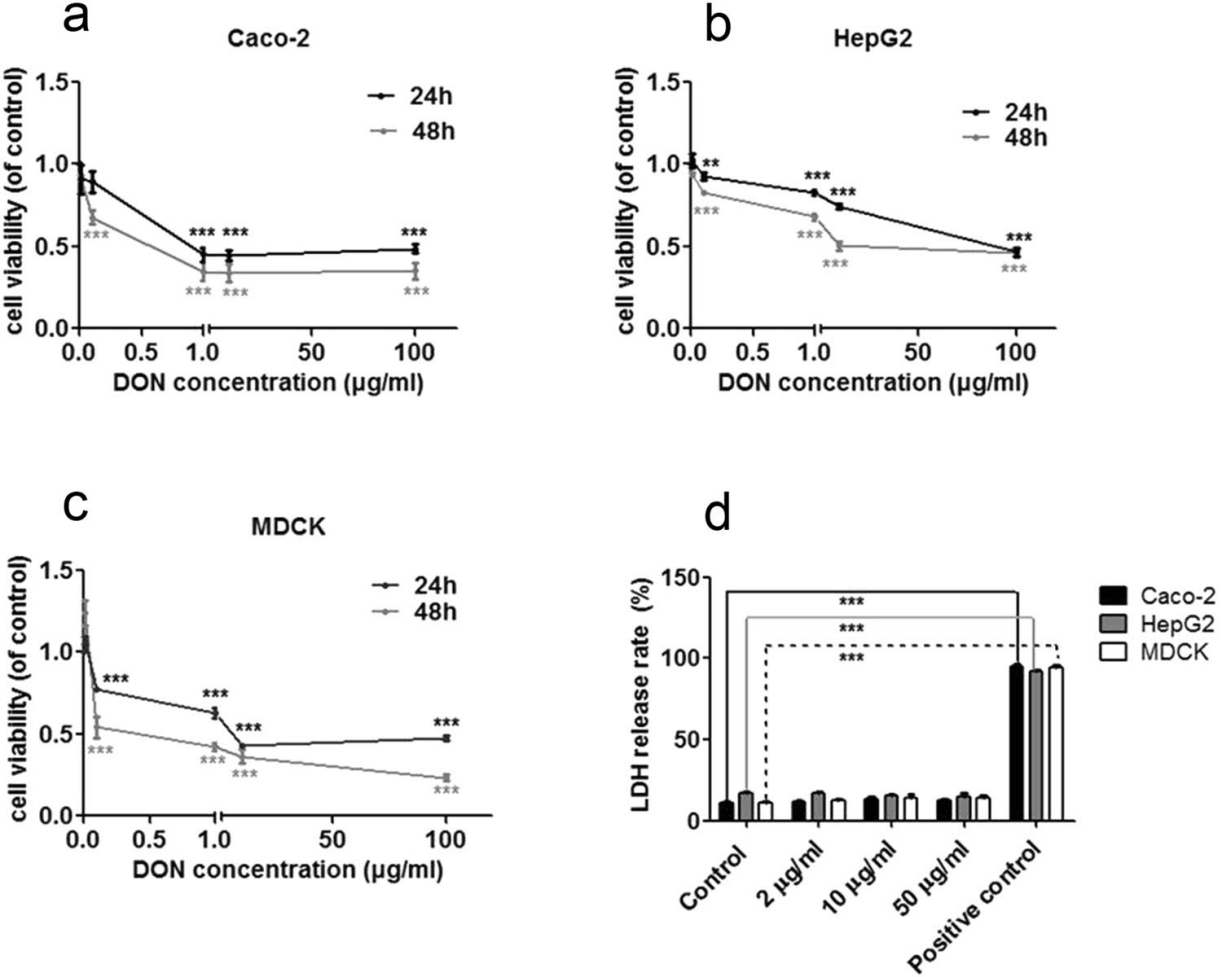
Cytotoxicity of DON toward Caco-2, HepG2 and MDCK cells was measured using the MTT assay and LDH assay. (**a**,**b** and **c**) The viability of Caco-2, HepG2 and MDCK cells was measured using the MTT assay after exposure to different concentrations of DON (0.01, 0.1, 1, 10, 100 μg/ml). (**d**) Rate of LDH release by Caco-2, HepG2 and MDCK cells exposed to 2 μg/ml to 50 μg/ml DON for 24 h. Nine percent Triton X-100 was used as the positive control. The data are presented as the mean values ± standard deviations (SD) (n = 5). Significant differences between the positive control and control group are indicated by **P < 0.01 and ***P < 0.001.

The MTT (3-(4,5-dimethylthiazol-2-yl)-2,5-diphenyltetrazolium bromide) assay and lactate dehydrogenase (LDH) assay were performed in Caco-2, HepG2 and MDCK cells to assess DON toxicity in different cell lines. After 24 and 48 h, DON induced toxicity in different cells in a dose- and time-dependent manner in the MTT assay (Fig. 1a–c). Additionally, the mean IC50 values for Caco-2, HepG2 and MDCK cells were approximately 0.5, 5, and 0.1 μg/ml, respectively (Fig. 1a–c), indicating that DON exerts obvious toxic effects on the proliferation of the tested cell lines. Compared to the nonionic surfactant Triton X-100 (9% w/v), no obvious damage to the membrane integrity was observed in DON-treated Caco-2, HepG2 and MDCK cells, as showed in the LDH release assay (Fig. 1d). Based on these results, the cytotoxic effects of DON on mammalian cells were not due to its effect on cell membrane integrity.

### Transport Modes and Sites of DON Accumulation

We first optimized the HPLC-UV and ELISA detection methods to fulfill different requirements for detecting the amount of DON in cells. The limit of HPLC-UV in detecting DON concentrations was less than 20 ng/ml, and the limit of ELISA was less than 1 ng/ml. HPLC-UV and ELISA exhibited satisfactory linear responses from 10 ng/ml to 10 μg/ml (r = 0.9997) and from 1 ng/ml to 81 ng/ml (r = 0.97), respectively. The average recoveries of DON for both test methods at different concentrations were greater than 90% (Supplementary Table S1).

**Figure 2:**
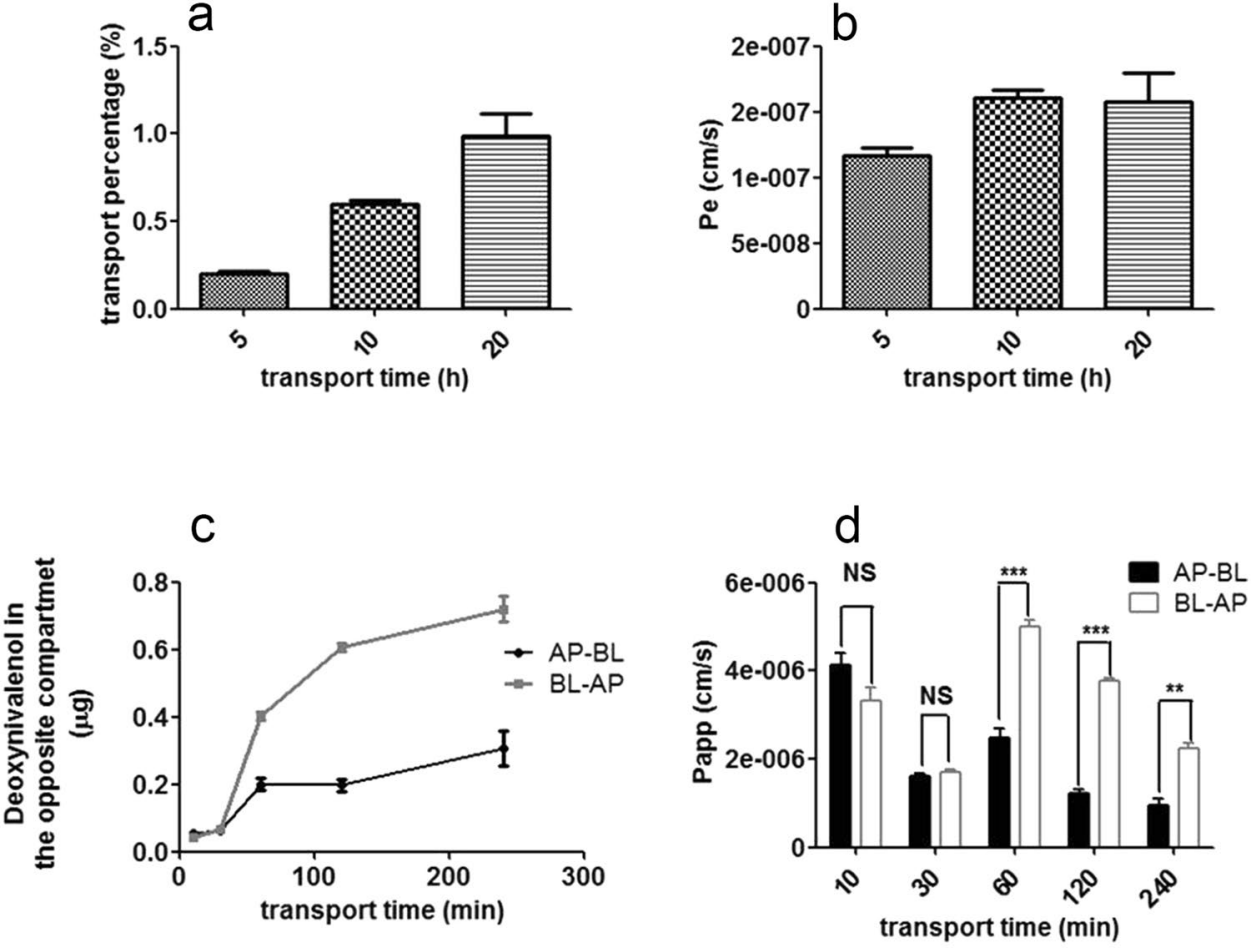
Analysis of the mechanisms of DON transport using the PAMPA and Transwell models. (**a**) The percentage of DON in the acceptor well of the PAMPA assay after 5, 10 and 20 h incubations with 10 μg/ml DON at 25 °C. (**b**) Permeability (Pe) (cm/s) observed after 5, 10 and 20 h. (**c**) Kinetics of the transepithelial flux of 10 μg/ml DON through a monolayer of MDCK cells in the Transwell model in the AP-BL and BL-AP directions. The data are presented as the mean values ± SD (n = 3). (**d**) Apparent permeability coefficients (P_*app*_) of DON in the Transwell model. Student’s t-test was used to analyze differences, and statistically significant differences in these values between the AP direction and the BL direction are indicated. **P < 0.01, ***P < 0.001. Abbreviations: AP, apical; BL, basolateral; NS, no significant difference.

PAMPA was used to determine whether the transepithelial transport of DON in mammalian cells occurs via simple diffusion (Supplementary Fig. S3). Less than 1% of DON was transferred to the opposite well (Fig. 2a). The mean permeability (*Pe*) values of DON were 1.21 × 10^−7^, 2.02 × 10^−7^, and 1.93 × 10^−7^ cm/s after 5, 10 and 20 h, respectively, at room temperature (Fig. 2b). These values were far lower than 10^−6^ cm/s, the value considered to represent high permeability in passive transport. However, the concentrations of T-2 toxin (positive control) in donor and acceptor wells were not significantly different after 5, 10 or 20 h (Supplementary Fig. S4). Thus, the main transport mode of DON is not simple diffusion.

A Transwell model with MDCK cells was used to mimic the uptake and efflux process in intestinal cells and further analyze DON transport. DON was rapidly transferred to the opposite well and reached the maximum value within 240 minutes in both the absorption (AP-BL) and efflux (BL-AP) directions in the Transwell model using MDCK cells (Fig. 2c). However, the apparent permeability coefficient (*P*_*app*_) of fluorescein, which is transported via the paracellular pathway, was less than 1 × 10^−6^ cm/s, showing that the Transwell model used in the tests worked well. Nevertheless, the permeability coefficient in the BL-AP direction was greater than that in the AP-BL direction, suggesting that DON uptake and efflux are asymmetric (Fig. 2c and d). Based on these results, transport proteins may participate in DON uptake and efflux^19^.

Because DNA and mitochondrial damage were reported in several cell types^10^, we further analyzed whether DON accumulated in the nucleus and mitochondria. We separated the nucleus and mitochondria using previously described methods^23, 24^ Interestingly, approximately 25% of DON was detected in the nucleus, but no DON was detected in mitochondria, suggesting that the mitochondria may be the main target of DON.

### DON Uptake and Efflux in Caco-2 Cells

**Figure 3:**
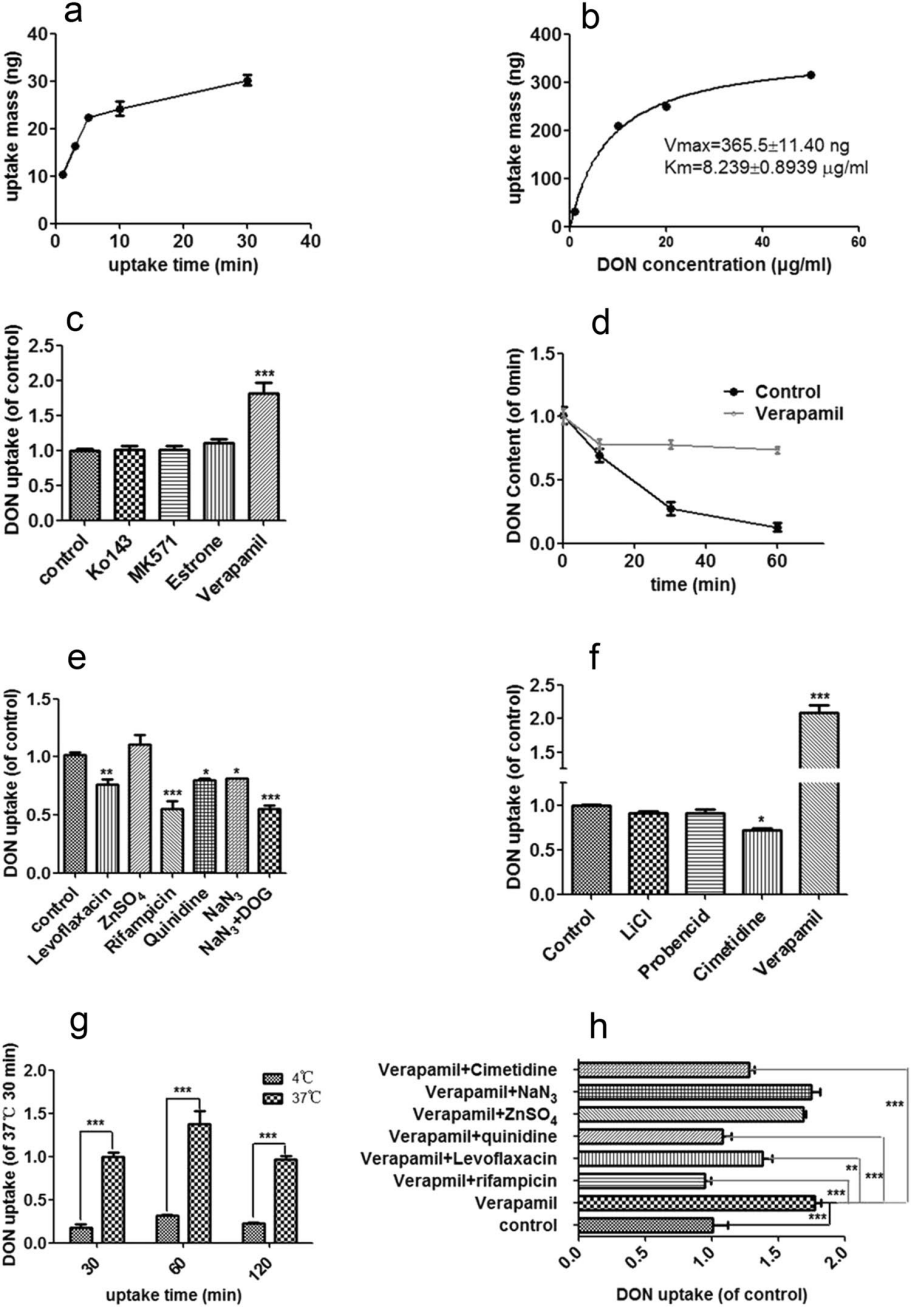
DON uptake and efflux in Caco-2 cells. (**a**) Time course of the transport of 5 μg/ml DON in Caco-2 cells (ELISA). (**b**) Effects of the DON concentration (0.5, 2, 10, 50 μg/ml) on transport at 3 min (ELISA). (**c**) Effects of inhibitors of efflux transporters on the transport of 5 μg/ml DON. Cells were pretreated with Ko143 (BCRP inhibitor, 10 μM), MK571 (MRP2 inhibitor, 50 μM), Estrone (ABCC1 inhibitor, 50 μM), and verapamil (P-glycoprotein inhibitor, 100 μM) for 30 min and then incubated with 5 μg/ml DON. (**d**) DON accumulation over time in verapamil-treated cells. (**e**) Effects of inhibitors of various highly expressed uptake transporters, including OCTN (levoflaxacin, 2 mM), OATP (rifampicin, 100 μM), PMAT (quinidine, 100 μM), PEPT (ZnSO_4_, 1 mM) and metabolic inhibitors (2 mM NaN_3_ and 50 mM 2-DOG). (**f**) Effects of inhibitors of various uptake transporters expressed at low levels, including OAT (probencid, 2 mM) and OCT (cimetidine, 2 mM). (**g**) Effects of temperature on the accumulation of 5 μg/ml DON. (**h**) Effects of combinations of uptake transporter inhibitors and verapamil. All data are presented as the mean values ± SD (n = 3) and represent the DON contents in the cells, which are normalized to the total protein mass. Abbreviations: 2-DOG, 2-deoxy-d-glucose; PMAT, plasma membrane monoamine transporter; PEPT, peptide transporters.

The intestine is the major absorptive organ in animals. Therefore, DON uptake and efflux were further characterized in a model intestinal cell line, Caco-2 cells. The concentration of DON in Caco-2 cells was first analyzed after a 1–30 min incubation. DON accumulated in Caco-2 cells through a linear process from 3 to 10 min and reached a steady state after 20 min (Fig. 3a). The whole-cell proportion of DON compared with the initial DON content is shown in Supplementary Fig. S5, indicating that a very low percentage of DON accumulated in whole cells. The effects of the DON concentration on the initial rate of DON uptake were studied further. DON accumulation was saturated at a high concentration, and the kinetic parameters were a V_max_ of 365.5 ± 11.40 ng and a Km of 8.239 ± 0.8939 μg/ml (Fig. 3b), indicating a carrier-mediated process was responsible for DON uptake and efflux.

Various transporter inhibitors were used to identify potential transporters involved in DON uptake and efflux. Regarding efflux transporters, DON accumulation in Caco-2 cells was strongly inhibited by a P-glycoprotein inhibitor (Verapamil), whereas other inhibitors, including an ABCC1 inhibitor (Estrone), a multidrug resistance-associated protein 2 (MRP2) inhibitor (MK571) and a breast cancer resistance protein (BCRP) inhibitor (Ko143), had no significant effect (Fig. 3c). The rate of DON efflux in the verapamil-treated group was much slower than the rate observed in the control group (Fig. 3d). Thus, P-glycoprotein may be the major efflux transporter of DON in Caco-2 cells.

DON uptake was strongly inhibited by an OATP inhibitor (Rifampicin), whereas other inhibitors, including an organic cation transporter novel (OCTN) inhibitor (Levoflaxacin) and a plasma membrane monoamine transporter (PMAT) inhibitor (Quinidine), had a slight influence, and a peptide transporter (PEPT) inhibitor (ZnSO_4_) had no effect (Fig. 3e). The effects of inhibitors of transporters expressed at low levels in intestinal cells, including OAT inhibitors (LiCl-replaced buffer, probencid) and OCT inhibitors (cimetidine), were also assessed but did not show a significant influence (Fig. 3f). Moreover, DON uptake was strongly inhibited by metabolic inhibitors (NaN_3_ and NaN_3_ + 2-DOG), indicating that the process was energy-dependent (Fig. 3e). The effects of inhibitors of uptake transporters were further confirmed by the combined application of uptake inhibitors with verapamil (Fig. 3h). Furthermore, DON accumulation in Caco-2 cells was strongly inhibited by 30, 60 and 120 min incubations at 4 °C (Fig. 3g). Thus, DON uptake and efflux are carrier-mediated processes in Caco-2 cells, and P-glycoprotein and OATP may be the major efflux transporter and uptake transporter for DON in Caco-2 cells, respectively.

### DON Uptake and Efflux in MDCK and HepG2 Cells

**Figure 4:**
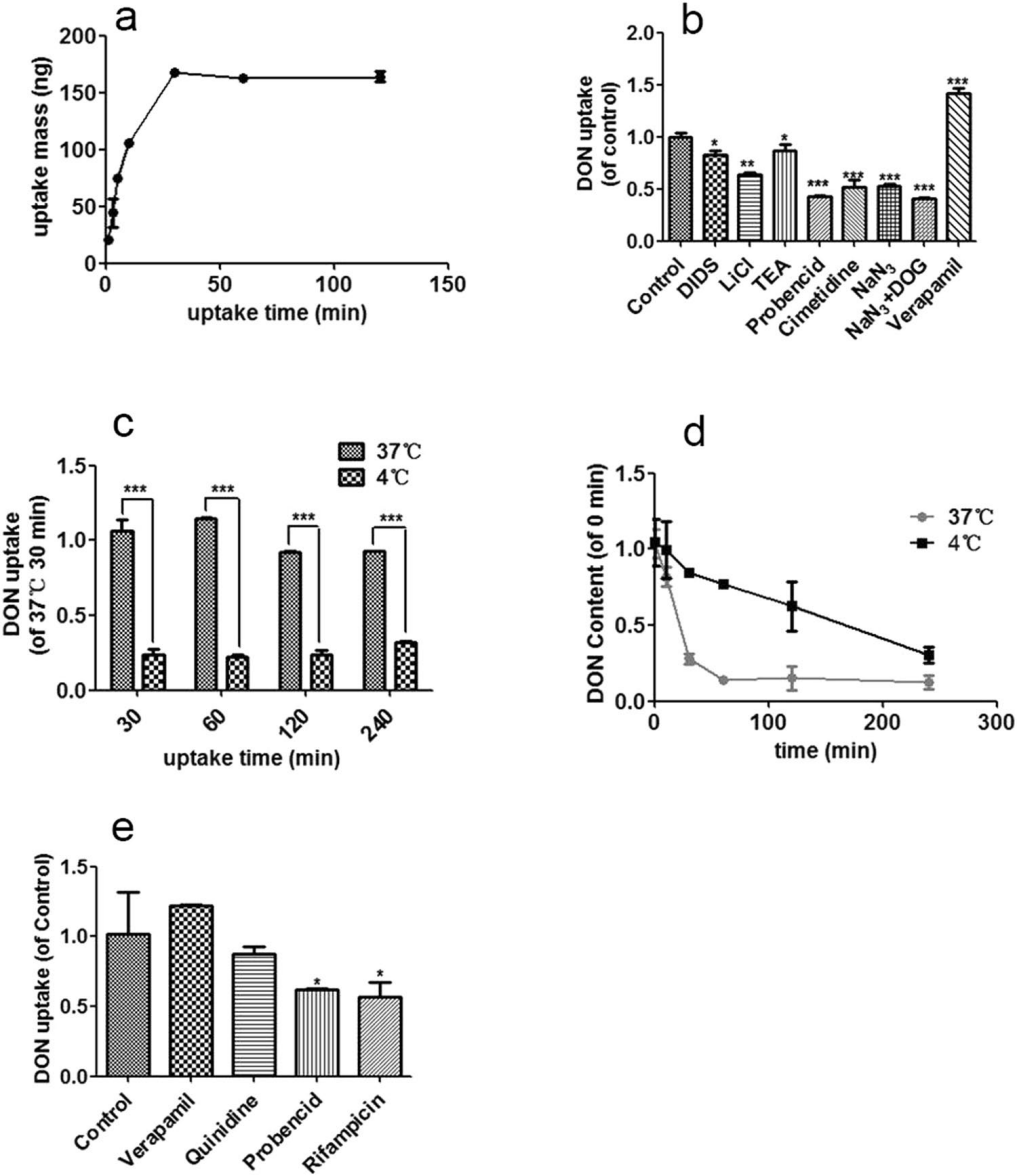
Characterization of DON transport in MDCK cells and HepG2 cells. (**a**) Time course of the transport of 20 μg/ml DON in MDCK cells; the data are presented as the mean values ± SD (n = 3) and are normalized to total protein concentrations; the total protein concentration measured at 1 min is set to 1. (**b**) Effects of OAT inhibitors (2 mM DIDS, 2 mM probencid, and 2 mM cimetidine), OCT inhibitors (2 mM TEA and 2 mM cimetidine), metabolic inhibitors (2 mM NaN_3_, 2 mM NaN_3_ + 50 mM 2-DOG) on the uptake of 20 μg/ml DON in MDCK cells within 10 min (n = 3). (**c**) Temperature-dependent uptake of 20 μg/ml DON in MDCK cells. (**d**) Temperature-dependent efflux of DON (n = 3) from MDCK cells. (**e**) Effects of the OAT inhibitor (100 μM probencid), OCT inhibitor (100 μM quinidine), OATP inhibitor (100 μM rifampicin) and P-glycoprotein inhibitor on the uptake of 5 μg/ml DON in HepG2 cells within 5 min (n = 3). Abbreviations: DIDS, 4,4′-diisothiocyanatostilbene-2,2′-disulfonic acid; TEA, tetraethylammonium chloride.

**Figure 5:**
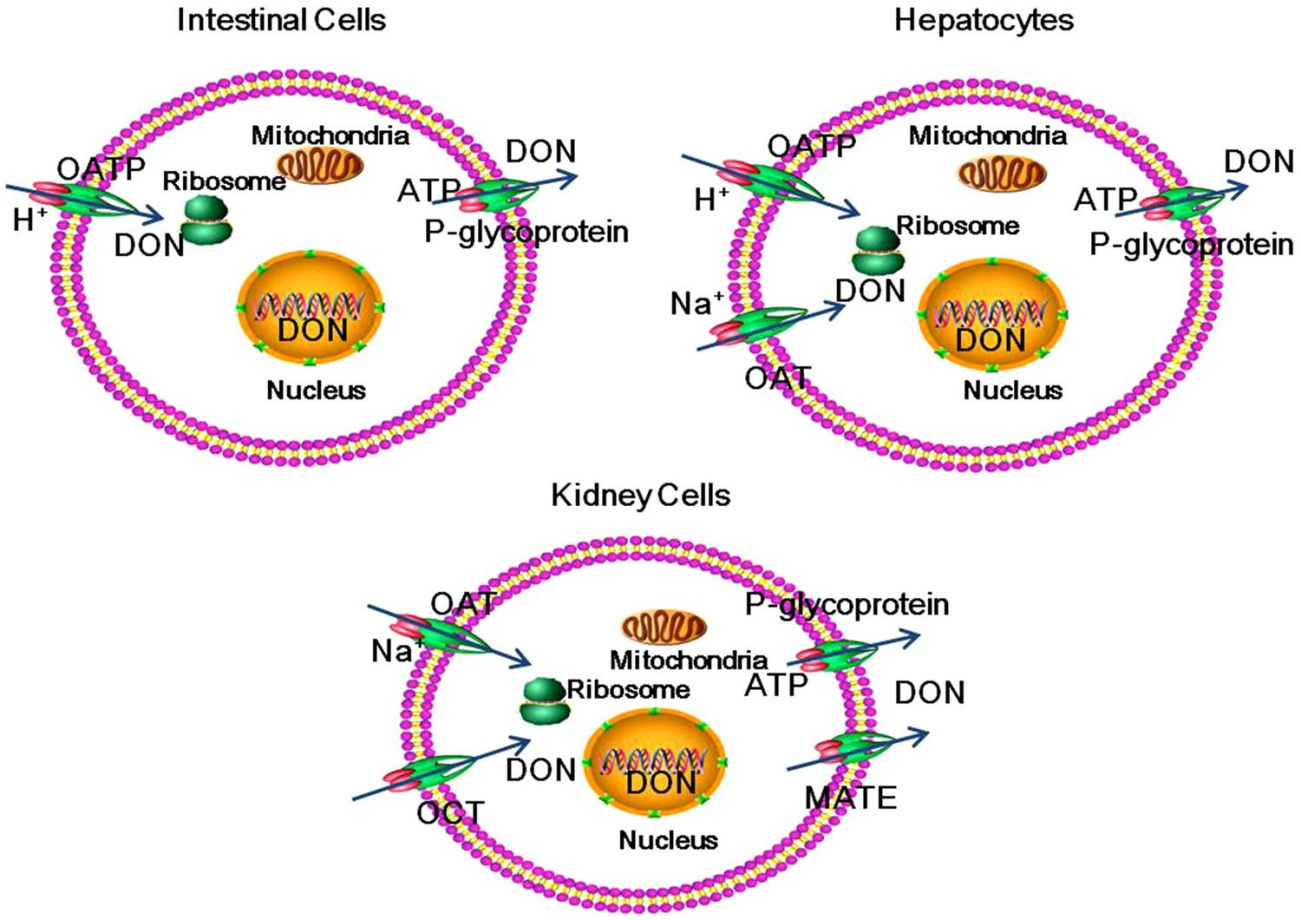
Postulated mechanisms of DON transport in mammalian cells. DON uptake and efflux is carrier-mediated and energy-dependent. P-glycoprotein is the major efflux transporter. OATPs, OATs and OATPs, and OATs and OCTs are the major uptake transporters of DON in intestinal cells, hepatocytes and kidney cells, respectively. Abbreviations: OATs, organic anion transporters; OCTs, organic cation transporters; OATPs, organic anion transporting peptide; MATEs, multidrug and toxin extrusion proteins.

The kidney and liver are the major target organs of DON^25, 26^. Because OATs and OCTs are expressed at higher levels in hepatocytes and kidney cells than in intestinal cells^27^, DON uptake and efflux were further investigated in the model cell lines, MDCK and HepG2 cells, to illustrate whether OATs and OCTs participate in the DON uptake process. Similar to Caco-2 cells, DON was also rapidly absorbed by MDCK cells and accumulated through a linear process from 3 min to 10 min (Fig. 4a); thus, the uptake was assessed from 3 to 10 min in the subsequent experiments. DON uptake by MDCK cells was strongly inhibited by OAT inhibitors (4,4′-diisothiocyanatostilbene-2,2′-disulfonic acid (DIDS), probencid, and LiCl-replaced buffer) and OCT inhibitors (tetraethylammonium chloride (TEA) and cimetidine) at both high and low concentrations (Figs 4b and S6). Similarly, DON uptake also depended on temperature and metabolism (Fig. 4b and c), indicating that the process depended on a carrier and energy. Concerning efflux in MDCK cells, P-glycoprotein was also the major efflux transporter (Fig. 4b). Interestingly, DON efflux in MDCK cells was strongly affected by temperature, indicating a carrier-dependent efflux process (Fig. 4d). The efflux carriers may include P-glycoprotein and multidrug and toxin extrusion proteins (MATEs) because the efflux process was also inhibited by the MATE inhibitor cimetidine (Supplementary Fig. S7). In HepG2 cells, DON uptake was also notably inhibited by OATP and OAT inhibitors (Rifampicin and Probencid, respectively) (Fig. 5e). Moreover, an OCT inhibitor (Quinidine) and a P-glycoprotein inhibitor (Verapamil) had slight effects on transport (Fig. 5e). In summary, P-glycoprotein was the major efflux transporter in MDCK and HepG2 cells, and both OAT and OCT are the major uptake transporters in MDCK cells and HepG2 cells; OATPs also participate in DON uptake in HepG2 cells.

## Discussion

DON is obviously toxic to mammalian cells due to its effects on cell proliferation^28^. Because DON toxicity depends on the dose and length of exposure (Fig. 1a–c), its toxicity was highly correlated with its accumulation in cells. However, the mechanism by which DON accumulates in cells remains unclear. In this study, DON uptake and efflux were extensively studied in Caco-2, MDCK and HepG2 cells.

Based on our results, we concluded that DON transfer is a carrier-mediated and energy-dependent process, consistent with the rapid absorption characteristics in mammalian cells^29^. First, DON is a small molecule with a molecular weight of less than 300 Daltons (Supplementary Fig. S1); thus, the possibility of bulk-phase endocytosis/pinocytosis is very low. Second, because DON contains three hydroxyl groups, it is a water-soluble molecule; thus, its transfer via free diffusion is unlikely. Third, free diffusion is not a major transfer mode for DON, according to the values of *Pe* and *Papp* (Fig. 2b and d). Finally, and most importantly, uptake of DON occurs in an energy-, temperature-, and concentration-dependent manner in Caco-2 and MDCK cells (Figs 3e,g and 4b and c). However, previous reports showed that DON entered cells via a paracellular pathway or free diffusion in Caco-2 cells^1–4, 16, 17^. We suggest this may be caused by the different experimental conditions used. The incubation time of DON with Caco-2 cells is longer than that used in our study. The long incubation may cause severe damage to cell membranes and DON can readily enter cells in damaged cells. The concentration of DON may also have a large effect on the transfer characteristics of DON. In those reports showing a paracellular pathway, or free diffusion of DON, the concentrations of DON were usually at high concentrations and significant cell damage was observed^12, 15–17, 30^. In our study, a short incubation time and low concentration of DON were used, and clearly showed that transporters participated in the DON transport (Figs 2, 3 and 4). We demonstrated for the first time that DON accumulates in the nucleus but not the mitochondria. DON has been shown to induce DNA damage in several cell types^31^. DON accumulation in the nucleus may partially explain the DNA damage induced by DON.

Cancer cells have been shown to be more resistant to various drugs than normal cells^27^, but the mechanism of resistance is unclear. As shown in this study, DON is mainly exported by P-glycoprotein in Caco-2, MDCK and HepG2 cells, of which Caco-2 and HepG2 are cancer cells (Figs 3c and 4b). DON efflux has been shown to be mediated by both P-glycoprotein and ABCC2 transporters^17^, which is slightly different from our results. However, P-glycoprotein was shown to be the major efflux protein for DON in both studies. Because the expression level and activity of P-glycoprotein are usually higher in cancer cells than in normal cells^32^, we hypothesized that these changes may be one possible reason why cancer cells are more resistant to DON than normal cells.

This present report is the first reporting transporters participating in DON uptake. Studies using the transporter inhibitors showed that OATs, OCTs and OATPs were the major transporters in different cell lines. OATs and OATPs are sodium-dependent transporters and hydrogen ion-dependent transporters, respectively, and OCTs are facilitated transporters^33, 34^. Thus, DON uptake occurs through two pathways, active transport and facilitated diffusion. Interestingly, the transporters involved in DON uptake differ in different cell types (Figs 3e and 4b), possibly due to the different expression levels of transporters in different tissues. OATPs are mainly expressed in intestinal cells and hepatocytes, whereas OCTs and OATs are mainly expressed in hepatocytes and kidney cells^33^. The differences in expression among the uptake transporters may explain the differences in the absorption, distribution and excretion of DON in different tissues. The over-expression of some potential transporters should be useful to identify which subtypes of these transporters are critical for DON uptake in future studies.

In summary, using PAMPA, Transwell membrane models and transporter inhibitors, a carrier-mediated and energy-dependent mechanism of DON transport was reported. P-glycoprotein is the major efflux transporter in all three tested cells, whereas OATPs, OATs and OATPs, and OATs and OCTs are the major uptake transporters of DON in intestinal cells, hepatocytes and kidney cells, respectively. These results will help us understand the mechanisms of DON toxicity and provide a theoretical basis for reducing DON-induced injury by reducing uptake and increasing efflux.

## Methods

### Cell culture

All cell lines, including Caco-2, MDCK and HepG2 cells, were grown in Dulbecco’s Modified Eagle’s Medium supplemented with 4.5 g/l D-glucose (Invitrogen, Carlsbad, CA) and 10% fetal bovine serum (BI, Israel) without any antibiotics in 100 × 10 mm cell culture dishes (Eppendorf, Germany) at 37 °C in a 5% carbon dioxide in air atmosphere. When the cells reached 80–90% confluence, they were plated on 6-well dishes or 96-well dishes at a density of 1.0 × 10^5^ cells/cm^2^ (Eppendorf). The culture medium was changed every 2 days for Caco-2, HepG2 and MDCK cells. All chemicals were obtained from Sigma (St. Louis, MO), unless indicated otherwise.

### PAMPA

A pre-coated PAMPA (Cat. No. 353015, Corning Costar, Acton, Massachusetts) was used as an *in vitro* model of passive, transcellular permeation^35^. Prior to use, the pre-coated PAMPA system was warmed to room temperature for at least 30 min. Next, 300 μl of compound in HBSS buffer (containing 8 g/l NaCl, 0.4 g/l KCl, 0.1 g/l MgSO_4_ · 7H_2_O, 0.1 g/l MgCl_2_ · 6H_2_O, 0.06 g/l Na_2_HPO_4_ · 2H_2_O, 0.06 g/l KH_2_PO_4_, 1.0 g/l D-glucose, 0.14 g/l CaCl_2_, and 0.35 g/l NaHCO_3_, adjusted to pH 7.4) and 200 μl of HBSS buffer were added to the receiver plate (donor wells) and filter plate (acceptor well), respectively. The filter was then placed on the receiver plate by slowly lowering the pre-coated PAMPA plate until it sat on the receiver plate. The assembly was incubated at 25 °C for 5, 10, 20 hours without shaking. The pre-coated PAMPA plate and receiver plate were then separated. The concentrations of the compound in both plates were determined using HPLC-UV methods, and DON permeability was calculated using equation (1).

### Methods for determining the DON and T-2 toxin concentrations

DON concentrations were analyzed using the Waters e2695 separation module system combined with a 2489 UV/Vis detector. An Agilent TC-C18 column (250 × 4.6 mm, 5 μm) was used to separate DON. An isocratic elution system with 85% mobile phase A and 15% mobile phase B was used, in which mobile phase A was water and mobile phase B was acetonitrile. The flow rate was 0.8 ml/min, and the injection volume was 50 μl. The determination wavelength was set at 218 nm. All data were obtained using HPLC-UV, unless indicated otherwise.

ELISA kits for DON and T-2 were purchased from Wuhan Huamei Biotech Company (Wuhan, PR China). Before use, all reagents in the kits and samples were placed in the laboratory at room temperature. Next, 50 μl of standard or sample was added to each well, followed by 50 μl of HRP-conjugate and 50 μl of antibody. The microtiter plate was then covered with a new adhesive strip and the contents were mixed well and incubated for 15 min or 30 min for DON or T-2, respectively. Each well was aspirated and washed, and then the process was repeated four times. Next, 100 μl of 3,3′,5,5′-tetramethylbenzidine (TMB) substrate was added to each well, and then the contents were mixed well. The plate was incubated for 5 min or 15 min for DON or T-2, respectively, protected from light. Fifty microliters of stop solution were added to each well and the plate was tapped gently to ensure thorough mixing. The optical density of each well was detected at 450 nm within 5 min using a microplate reader. The ranges of DON and T-2 concentrations determined using this assay were 1–81 ppb and 0.15–4.05 ppb, respectively.

### Cytotoxicity assays - MTT assay

The cytotoxic effects of DON on various cell lines were determined using the MTT assay. The MTT assay was performed as previously described^18^. Briefly, 24 h after seeding 1.0 × 10^4^ cells in each well of 96-well plates with at least 100 μl of medium, the cells were incubated with various concentrations of DON ranging from 0.01 μg/ml to 100 μg/ml for 24 h and 48 h. After incubation, a 0.5 mg/ml MTT solution was added to each well. After a 4 h incubation at 37 °C in the dark, the solution was aspirated, and the formazan crystals were dissolved in DMSO. Next, the optical density of each well was determined at 595 nm using a microplate reader.

### LDH assay

Direct cytotoxicity of DON was determined by measuring extracellular LDH release^36, 37^. The underlying mechanism of this assay is that LDH catalyzes the conversion of lactic acid to pyruvic acid. Pyruvic acid reacts with 2,4-dinitrophenylhydrazine to generate dinitrophenylhydrazone pyruvate, which produces a brown-red color in alkaline solution. Enzyme activity was measured using a colorimetric method. LDH activity was determined according to the brochure provided by the Nanjing Jiancheng Bioengineering Company (Nanjing, China).

### Transwell

Transwell studies were performed using MDCK cells. MDCK cells were seeded on 0.4 μm Transwell filters in 12-well plates (Corning Costar, Acton, Massachusetts). The monolayers were pre-incubated in HBSS for 30 min at 37 °C. The volumes in the apical (AP) and basolateral (BL) chambers were 0.5 ml and 1.5 ml HBSS (pH 7.4), respectively. DON (10 μg/ml) in HBSS was added in the AP chamber and BL chamber to study AP-BL and BL-AP transfer. Samples were collected in the opposite compartment at 10, 30, 60, 120 and 240 min. The flux of 300 μg/ml fluorescein was measured to assess paracellular permeability^38^. Monolayers were excluded if the *P*_*app*_ of fluorescein in the receiver compartment exceeded 1.0 × 10^−6^ cm/s.

### Uptake studies

Cells at 90–100% confluence were washed twice with PBS (materials) and incubated with warmed HBSS for 30 min at 37 °C. Next, the test solution containing DON was added. At the end of the incubation, the test solution was aspirated and the cells were washed with ice-cold PBS three times. The effects of well-known OATP (Rifampicin), OAT (Probencid and DIDS), OCT (Cimetidine, Quinidine, and TEA), OCTN (Levoflaxacin), P-glycoprotein (Verapamil), MRP2 (MK571), BCRP (Ko143), and PEPT (ZnSO_4_) inhibitors on uptake were measured. Cells were pre-incubated with the inhibitors for 30 min, and various concentrations of DON combined with the inhibitors were added to the medium. Ko143, MK571 and Rifampicin were obtained from Sigma (St. Louis, MO), and the other chemicals were obtained from TCI (Tokyo, Japan). DON uptake was quantified using a previously described method^12^. The concentrations of the inhibitors used in this study were based on the literature and pretests^16, 18, 20^.

### Sample preparation

Approximately 200 μl of sample were measured into a 2 ml centrifuge tube. Next, 500 μl of acetic ether were added and the mixture was incubated for 5 min. Following centrifugation at 5000 × g for 5 min at 4 °C, the supernatant was collected and evaporated to dryness under a stream of nitrogen in a water bath at 60 °C. The residue was reconstituted with 200 μl of solution (15% acetonitrile-85% water), vortexed for 5 min, and 50 μl of solution was transferred to autosampler vials for HPLC-UV analysis or ELISA.

### Data analysis

All statistical procedures were performed using SPSS 17.0 software for Microsoft Windows. Statistically significant differences were determined using one-way analysis of variance (ANOVA) followed by Bonferroni’s multiple comparison tests for more than two groups and Student’s test for two groups (two tailed). The results were expressed as the means ± SEM. Statistical significance was defined as *P < 0.05, **P < 0.01 or ***P < 0.001.

The permeability values (*P*_e_) of the PAMPA models were obtained using the following equation^35^:

1 $${P}_{e}=\frac{-\,\mathrm{ln}[1-{C}_{A}(t)/{C}_{equilibrium}]}{{A}^{\ast }(1/{V}_{D}+1/{V}_{A}{)}^{\ast }t}$$

Mass Retention: R = 1 − [C_D_(t)*V_D_ + C_A_(t)*V_A_]/(C_0_*V_D_)

C_0_: initial concentration of the compound in the donor well (mM); C_D_(t): concentration of the compound in the donor well at time t (mM); C_A_(t): concentration of the compound in the acceptor well at time t (mM); V_D_: donor well volume = 0.3 ml; V_A_: acceptor well volume = 0.2 ml; C_equilibrium_ = [C_D_(t)*V_D_ + C_A_(t)*V_A_]/(V_D_ + V_A_); A: filter area = 0.3 cm^2^; t: incubation time = 18000 s (=5, 10, or 20 h).

The apparent permeability coefficients (*P*_app_) of the Transwell models were calculated using the following equation^18^:

2 $${P}_{app}=\frac{dQ/dt}{A{C}_{0}}$$

where *dQ*/*dt* is the slope of the cumulative amount of compound transported throughout the study. A is the area of the inserts and *C*_0_ is the starting concentration.

## Electronic supplementary material

Supplementary Information

## References

[1] Placinta C, D’mello J, Macdonald A (1999). A review of worldwide contamination of cereal grains and animal feed with Fusarium mycotoxins. Anim Feed Ssi Tech.

[2] Organization, World Health. Selected mycotoxins: ochratoxins, trichothecenes, ergot. *Environmental Health Criteria* **105** (Geneva, 1990).

[3] Scott, P. The natural occurrence of trichothecenes. *Trichothecene mycotoxins: pathophysiologic effects* (ed. Beasley, V. R.) 1–26 (Baco Raton, 1989).

[4] International Agency for Research on Cancer. IARC Monographs of the evaluation of carcinogenic risks of Chemicals to human. Some Naturally-occuring Substances: Some Food items and Constituents, Heterocyclic Aromatic Amines and Mycotoxins. *International Agency for Research on Cancer* **56**, 245–395 (Lyon, 1993).

[5] Canady, R. *et al*. Deoxynivalenol. Safety evaluation of certain mycotoxins in food. Joint Expert Committee on Food Additives (JECFA). *WHO Food Additives Series* **47**, 419–555 (Geneva, 2001).

[6] Rotter BA (1996). Invited review: Toxicology of deoxynivalenol (vomitoxin). J Toxicol Env Heal A.

[7] Pestka JJ, Smolinski AT (2005). Deoxynivalenol: toxicology and potential effects on humans. J Toxicol Env Heal B.

[8] Pestka JJ (2010). Deoxynivalenol: mechanisms of action, human exposure, and toxicological relevance. Arch Toxicol.

[9] Maresca M (2013). From the gut to the brain: Journey and pathophysiological effects of the food-associated trichothecene mycotoxin deoxynivalenol. Toxins.

[10] Bensassi F (2012). Involvement of mitochondria-mediated apoptosis in deoxynivalenol cytotoxicity. Food Chem Toxicol.

[11] Miller JD, Ewen MA (1997). Toxic effects of deoxynivalenol on ribosomes and tissues of the spring wheat cultivars Frontana and Casavant. Nat Toxins.

[12] Akbari P (2014). Deoxynivalenol: a trigger for intestinal integrity breakdown. FASEB J.

[13] Awad WA, Zentek J (2015). The feed contaminant deoxynivalenol affects the intestinal barrier permeability through inhibition of protein synthesis. Arch Toxicol.

[14] Behrens M, Huwel S, Galla HJ, Humpf HU (2015). Blood-Brain Barrier Effects of the Fusarium Mycotoxins Deoxynivalenol, 3 Acetyldeoxynivalenol, and Moniliformin and Their Transfer to the Brain. PLoS One.

[15] Kadota T (2013). Comparative study of deoxynivalenol, 3-acetyldeoxynivalenol, and 15-acetyldeoxynivalenol on intestinal transport and IL-8 secretion in the human cell line Caco-2. Toxicol in Vitro.

[16] Sergent T (2006). Deoxynivalenol transport across human intestinal Caco-2 cells and its effects on cellular metabolism at realistic intestinal concentrations. Toxicol Lett.

[17] Videmann B, Tep J, Cavret S, Lecoeur S (2007). Epithelial transport of deoxynivalenol: involvement of human P-glycoprotein (ABCB1) and multidrug resistance-associated protein 2 (ABCC2). Food Chem Toxicol.

[18] Wang X (2014). High risk of embryo-fetal toxicity: Placental transfer of T-2 toxin and its major metabolite HT-2 toxin in BeWo cells. Toxicol Sci.

[19] Dean M, Hamon Y, Chimini G (2001). The human ATP-binding cassette (ABC) transporter superfamily. J Lipid Res.

[20] Tep J (2007). Transepithelial transport of fusariotoxin nivalenol: mediation of secretion by ABC transporters. Toxicol Lett.

[21] Artursson P, Palm K, Luthman K (2001). Caco-2 monolayers in experimental and theoretical predictions of drug transport. Adv Drug Deliver Re.

[22] Irvine JD (1999). MDCK (Madin–Darby canine kidney) cells: a tool for membrane permeability screening. J Pharm Sci.

[23] Lampl T, Crum JA, Davis TA, Milligan C, Moore VDG (2015). Isolation and functional analysis of mitochondria from cultured cells and mouse tissue. J Vis Exp.

[24] Abmayr, S. M., Carrozza, M. J. & Workman, J. L. Preparation of nuclear and cytoplasmic extracts from mammalian cells. *Current protocols in pharmacology Chapter 12*, Unit 12.1 (2006). 10.1002/0471141755.ph1203s35 22294172

[25] Yordanova J, Rosso OA, Kolev V (2003). A transient dominance of theta event-related brain potential component characterizes stimulus processing in an auditory oddball task. Clin Neurophysiol.

[26] Gouze M (2004). Individual and combined effects of low oral doses of deoxynivalenol and nivalenol in mice. Cell Mol Biol.

[27] Gottesman MM, Fojo T, Bates SE (2002). Multidrug resistance in cancer: role of ATP–dependent transporters. Nat Rev Cancer.

[28] Minervini F, Fornelli F, Flynn K (2004). Toxicity and apoptosis induced by the mycotoxins nivalenol, deoxynivalenol and fumonisin B 1 in a human erythroleukemia cell line. Toxicol in Vitro.

[29] Goyarts T, Dänicke S (2006). Bioavailability of the Fusarium toxin deoxynivalenol (DON) from naturally contaminated wheat for the pig. Toxicol Lett.

[30] Nielsen JK, Vikstrom AC, Turner P, Knudsen LE (2011). Deoxynivalenol transport across the human placental barrier. Food Chem Toxicol.

[31] Frankič T, Pajk T, Rezar V, Levart A, Salobir J (2006). The role of dietary nucleotides in reduction of DNA damage induced by T-2 toxin and deoxynivalenol in chicken leukocytes. Food Chem Toxicol.

[32] Cole S (1992). Overexpression of a transporter gene in a multidrug-resistant human lung cancer cell line. Science.

[33] Nigam SK (2015). What do drug transporters really do?. Nat Rev Drug Discov.

[34] Visentin M, Chang M-H, Romero MF, Zhao R, Goldman ID (2012). Substrate-and pH-specific antifolate transport mediated by organic anion-transporting polypeptide 2B1 (OATP2B1-SLCO2B1). Mol Pharm.

[35] Bermejo, M. *et al*. PAMPA—a drug absorption in vitro model: 7. Comparing rat in situ, Caco-2, and PAMPA permeability of fluoroquinolones. *Eur*. *J*. *Pharm*. *Sci* **21**, 429–441 (2004). 10.1016/j.ejps.2003.10.009 14998573

[36] Kao Y-Y, Chen Y-C, Cheng T-J, Chiung Y-M, Liu P-S (2012). Zinc oxide nanoparticles interfere with zinc ion homeostasis to cause cytotoxicity. Toxicol Sci.

[37] Lison D (2008). Nominal and effective dosimetry of silica nanoparticles in cytotoxicity assays. Toxicol Sci.

[38] Liu, F., Soares, M. J. & Audus, K. L. Permeability properties of monolayers of the human trophoblast cell line BeWo. *Am J Physiol-Cell Ph* **273**, C1596–C1604 (1997). 10.1152/ajpcell.1997.273.5.C1596 9374645

