# Supplementary material for: Carrier-Mediated and Energy-Dependent Uptake and Efflux of Deoxynivalenol in Mammalian Cells
Source: Sci Rep. 2017 Jul 19;7:5889. doi: 10.1038/s41598-017-06199-8 (PMC5517473; doi:10.1038/s41598-017-06199-8)
Supplement: Supplementary file 1 — Supplementary Information [file 41598_2017_6199_MOESM1_ESM.pdf]

Carrier-Mediated and Energy-Dependent Uptake and Efflux of Deoxynivalenol in Mammalian Cells

Authors: **Xiaoming Li, Peiqiang Mu, Jikai Wen and Yiqun Deng**

Supplementary Data

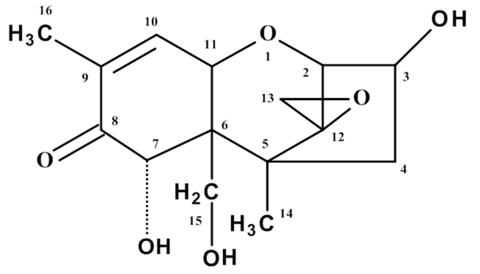

Supplemental Figure s1. Chemical structure of Deoxynivalenol (DON)

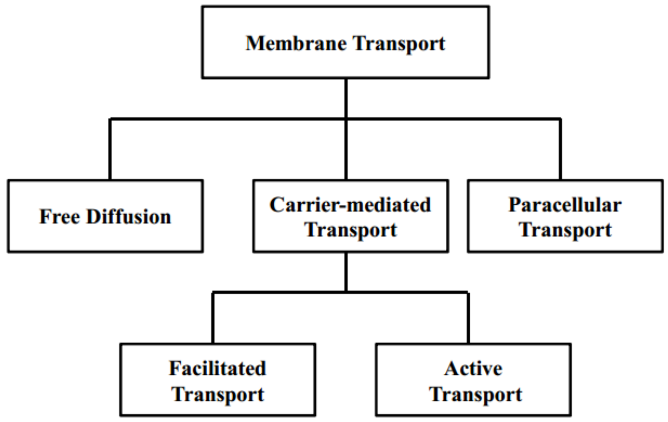

Supplemental Figure s2. Possible transport modes of the DON toxin in mammalian cells.

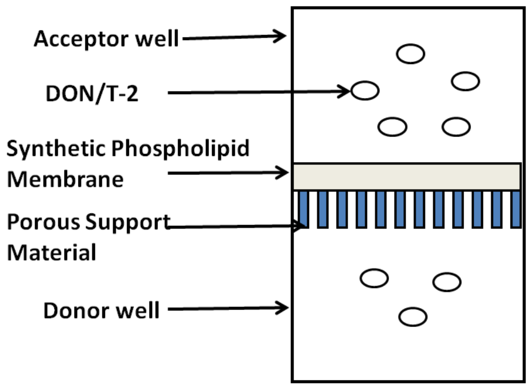

Supplemental Figure s3. Parallel Artificial Membrane Permeability (PAMPA) model.

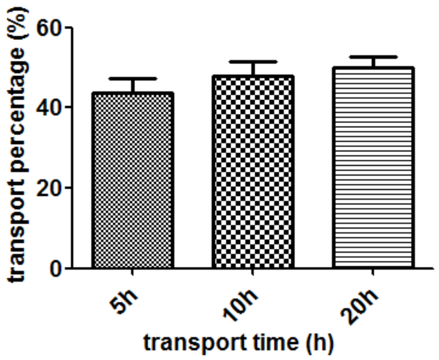

Supplemental Figure s4. Percentage of 1 μg/ml T-2 toxin transported in the PAMPA model.

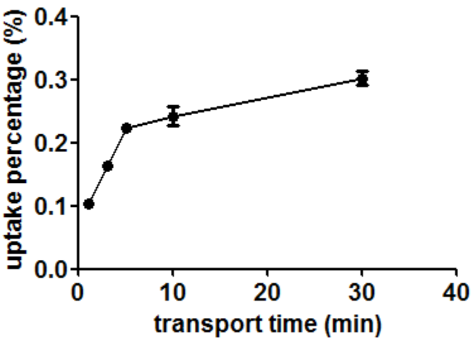

Supplemental Figure s5. Percentage of the DON content taken up by the whole cell compared with the total initial DON content in the medium. The initial DON concentration was 5 μg/ml.

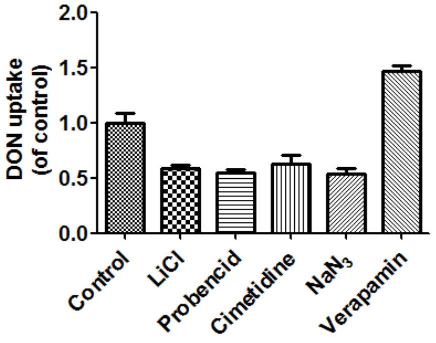

Supplemental Figure s6. Effects of various transporter inhibitors on the uptake of 5 μg/ml DON in MDCK cells within 10 min.

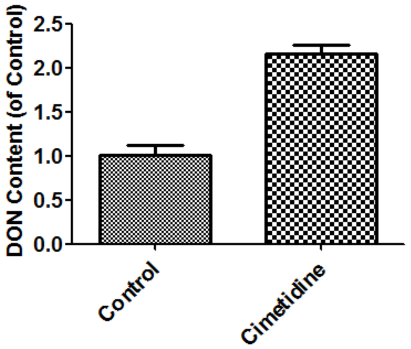

Supplemental Figure s7. Effect of multidrug and toxin extrusion proteins (MATE) inhibitor cimetidine on DON efflux from MDCK cells. MDCK cells were pre-incubated with 5 μg/ml DON, and the medium was replaced with HBSS buffer or HBSS buffer containing 2 mM Cimetidine after a 10 min incubation with DON to allow uptake; the DON content in the whole cells was measured after 10 min of efflux.

Supplemental Table s1. Accuracy and Precision of DON Concentrations Measured using Each Method at 3 Spiking Levels (Mean).

	Concentration (ng/ml)	Mean Recoveries SD (%)	RSD (%)
HPLC-UV Methods	10000	96.1	2.18
	1000	90.1	3.55
	100	91.3	4.49
ELISA Methods	50	94.1	3.72
	5	92.1	4.56
	1	93.1	4.72
